# Non-alcoholic fatty liver disease in underweight patients with inflammatory bowel disease: A case-control study

**DOI:** 10.1371/journal.pone.0206450

**Published:** 2018-11-14

**Authors:** Lisa C. Adams, Falk Lübbe, Keno Bressem, Moritz Wagner, Bernd Hamm, Marcus R. Makowski

**Affiliations:** Department of Radiology, Charité, Berlin, Germany; Medizinische Fakultat der RWTH Aachen, GERMANY

## Abstract

Non-alcoholic fatty liver disease (NAFLD) was shown to also occur in lean and underweight patients. So far, the prevalence of NAFLD in underweight individuals with and without inflammatory bowel disease (IBD) is insufficiently enlightened. In this cross-sectional age, gender and disease-matched case-control study, underweight patients (BMI<18.5 kg/m^2^) with inflammatory bowel disease (IBD), who underwent abdominal MRI at 1.5 T/3 T with fat-saturated fast-spin-echo imaging from 10/2005-07/2018 were analysed (control-to-case-ratio 1:1, n = 130). All patients were additionally investigated for duration, history of surgery, medical treatment, laboratory values, liver and spleen diameters. On MRI, liver fat was quantified by two observers based on the relative signal loss on T2-weighted fast spin-echo MR images with fat saturation compared to images without fat saturation. The prevalence of NAFLD/liver steatosis, defined as a measured intrahepatic fat content of at least 5%, was significantly higher in underweight IBD patients than in normal weight patients (87.6% versus 21.5%, p<0.001). Compared to the cases, the liver fat content of the controls was reduced by -0.19 units on average (-19%; 95%Cl: -0.20; -0.14). Similar results were obtained for the subgroup of non-IBD individuals (n = 12; -0.25 units on average (-25%); 95%Cl: -0.35; -0.14). Patients with extremely low body weight (BMI <17.5 kg/m^2^) showed the highest liver fat content (+0.15 units on average (+15%) compared to underweight patients with a BMI of 17.5–18.5 kg/m^2^ (p<0.05)). Furthermore, underweight patients showed slightly increased liver enzymes and liver diameters. There were no indications of significant differences in disease duration, type of medications or surgery between cases and controls and also, there were no significant differences between observers or field strengths (p>0.05). The prevalence of liver steatosis was higher among underweight IBD and non-IBD patients compared to normal weight controls. Also, underweight patients showed slightly increased liver enzymes and liver diameters, hinting at initial metabolic disturbances.

## Introduction

Non-alcoholic fatty liver disease (NAFLD) has increased over the past decades to become one of the most common causes of chronic liver disease in Western countries [1, 2]. It is generally considered a manifestation of the metabolic syndrome and associated with obesity or central adiposity [3]. Although metabolic risk factors are undoubtedly major causes for hepatic steatosis, it is known that it can also occur in normal weight and underweight patients [4]. With regard to malnutrition, it was observed in the context of the refeeding process, but also as a potential result from starvation-induced autophagy and metabolic dysfunction in the human liver [5–7]. However, so far, the prevalence of NAFLD in underweight individuals is insufficiently enlightened.

Malnutrition with significantly low weight has long been recognized as showing detrimental effects on health and different organ functions, generally being defined by a body mass index (BMI) < 18.5 kg/m^2^ [8, 9]. While malnutrition is often associated with cancer, AIDS, congestive heart failure or anorexia, it is also common in patients suffering from inflammatory bowel diseases (IBDs). IBDs are characterized by a chronic remitting inflammation of the gastrointestinal tract, encompassing Crohn’s disease (CD) and ulcerative colitis (UC) [10]. Malnutrition and cachexia are particularly common in patients with active Crohn’s disease, but nutritional deficiencies can also develop in patients with active ulcerative colitis [11]. The frequency reported for undernutrition in active IBD patients ranges from 25% to 69.9% and severe undernutrition was previously found in up to 31.6% of the patients [12]. Furthermore, IBD are frequently associated with extraintestinal manifestations, including the liver. For patients with IBD, the prevalence of NAFLD has been estimated between 8–40%, showing no difference between CD and UC [13–15]. So far, apart from bowel surgery and medication, especially metabolic risk factors and obesity were suggested to affect the presence of NAFLD [14, 15]. However, a recent study described NAFLD in nearly 40% of the IBD patients with a mean body mass index (BMI) of 21 kg/m^2^, but without examining the influence body weight [13].

The aim of this study was to test whether low weight is associated with steatosis on magnetic resonance (MR) imaging. To test this hypothesis, we conducted a cross-sectional age-, gender- and disease-matched retrospective case-control study, comparing the presence of NAFLD in IBD patients with significantly low weight to IBD patients with normal weight. In a smaller cohort, patients without systemic disease, but significantly low underweight, were compared to healthy patients with normal weight. To exclude potential confounders, we also assessed previously acknowledged risk factors for NAFLD, such as medical treatment or bowel surgery.

## Methods

This retrospective study was performed in accordance with the relevant guidelines and declarations and approved by the Institutional Review Board (Charité’s Ethics Commission, EA4/054/18), including a formal waiver of informed consent. For confidentiality and ethical considerations, the retrospective data used encrypted identification of the individuals, with the identification numbers of the individuals being transformed using random number strings. Medical records of underweight patients, who underwent diagnostic evaluation at our institution between October, 2005 and July, 2018, were reviewed. Indication for MRI were mainly evaluation and follow-up of inflammatory bowel disease, including e.g. detection of potential fistula and perianal inflammation or assessment of treatment response. In the twelve underweight patients without IBD and the corresponding controls, MRI was performed to exclude tumor disease or IBD (in view of a previous weight loss) or because of abdominal pain. In five cases, the primary indication for MRI was the detection of a possible liver disease.

For each of the included patients (n = 61), an age-, gender- and disease-matched control with normal weight was obtained (n = 61). The primary outcomes were the frequency of NAFLD/ degree of hepatic steatosis detected by MRI in patients with significantly low weight, compared to an age-, gender- and disease-matched group of patients with normal weight.

### Inclusion / Exclusion criteria and study groups

For the cases, inclusion criteria were all patients with (1) underweight (BMI < 18.5 kg/m^2^), who had received (2) abdominal MRI (including T2 HASTE sequences with and without fat saturation) at our institution. For the controls, inclusion criteria were patients with (1) normal weight (BMI of 20–24.9 kg/m^2^, according to the normal weight definition of the World Health Organization) and (2) MRI performed at our institution (same imaging criteria as for case groups). Exclusion criteria were patients with known viral hepatitis B and/or C infection, drug-abuse, alcohol-related liver disease and hereditary/metabolic liver diseases, such as hemochromatosis, alpha-1-antitrypsin deficiency, Wilson’s disease, autoimmune hepatitis, familial hypercholesterolemia, primary biliary cholangitis, primary sclerosing cholangitis or celiac disease [16, 17]. Patients receiving parental nutrition were also excluded. The assessment of alcohol consumption relied on prior clinical evaluation (history taking) and was considered significant, if at any time there was an indication of alcoholism, alcohol abuse or treatment of an alcohol-related diagnosis in the electronic medical records. In addition, medical files were searched for alcohol-associated diagnoses in the 5 years prior to the MRI examination.

The cases were divided into a first group who had received MRI at 1.5 T and a second group who had received MRI at 3 T. The 1.5 T group consisted of 25 patients (19 women, 6 men, average age 31.8 ± 9.8 years, average BMI 17.3 ± 1.5 kg/m^2^). Out of these, 24 patients were diagnosed with Crohn’s disease and 1 patient with ulcerative colitis. The 3 T group consisted of 36 patients (21 women, 15 men, average age 30 ± 8.1 years, average BMI was 17.1 ± 1.2 kg/m^2^), with 23 patients being diagnosed with Crohn’s disease, 5 patients with ulcerative colitis and 8 patients suffering from indistinct stomach pains without diagnosis of physical disorders.

The age-, gender- and disease-matched controls (control-to-case ratio 1:1) consisted of patients with normal weight, who were examined at 1.5 T (matched to first case group) or at 3 T (matched to second case group). For each patient in the two case groups, a control with normal BMI and matched age (± 2 years), gender and disease was obtained. For the 1.5 T control group. the average age was 32.0 ± 9.7 years and average BMI was 22.1 ± 1.5 kg/m^2^. For the 3T control group, the average age was 22.6 ± 9.1 years and average BMI was 22.6 ± 1.7 kg/m^2^.

Diagnoses of CD or UC were given after a combined evaluation of symptoms, abdominal imaging, endoscopy and histology. The diagnosis of NAFLD was made based on MRI of the liver, which included a T1 weighted sequence and T2-weighted sequences with and without fat saturation.

### Clinical study variables

Demographic and clinical data were collected and all of the included patients were examined by nutrition or IBD specialists. In patients with IBD, variables such as the type of IBD (CD/UC), surgical therapies (type of bowel loss) were considered. Furthermore, prior and current medical treatments were assessed, including systemic therapies with corticosteroids, 5-amino-salicylic agents (5 ASA) or immunomodulators (e.g. azathioprine. 6-mercaptopurine), which might promote the development of NAFLD, but also therapies with biological agents (TNF-α-inhibitors) or immunosuppressant medications such as cyclosporine without known effects on hepatic steatosis. In addition, laboratory values acquired around the date of the MRI (< 6 months) were extracted from the medical records. These included: Total bilirubin (mg/dL), albumin (Alb) (g/L), alanine aminotransferase (ALT) (U/L), aspartate aminotransferase (AST) (U/L), alkaline phosphatase (AP) (U/L) and gamma-glutamyltransferase (GGT) (U/L).

### MRI technique

MRI was performed on a 3 T scanner with 64 channels (Magnetom Skyra^™^, Siemens Healthcare, Germany) and on 1.5 T scanners with 16/48 channels (Magnetom Avanto/Aera, Siemens Healthcare, Germany). The patients in this study underwent a routine abdominal imaging protocol, including: axial T1 FLASH, axial/coronal T2 HASTE and axial/coronal T2 HASTE fatsat (see Table 1 for tabulated imaging parameters).

**Table 1:** Tabulated MRI parameters.

	1.5 T		3 T	
Type of acquisition	T2 HASTE* axial and coronary with/without fat saturation	T1 FLASH**	T2 HASTE*axial and coronary with/without fat saturation	T1 FLASH**
Repetition time, TR (ms)	1400	216	1600	153
Echo time, TE (ms)	91	4.76	94	2.46
Field of view (FOV)	360 × 360	360 x 360	340 x 340	380 x 380
Matrix size	256 x 256	320 x 320	320 x 320	320 x 320
Slice thickness (mm)	6	6	6	5
Pixel bandwidth (Hz/pixel)	698	140	710	270
Acquisition mode	2D	2D	2D	2D
Flip angle (°)	180	70	160	69
Voxel size	1.4x1.4x6.0	1.4x1.4x6.0	1.1x1.1x6.0	1.2x1.2x5.0
Fat saturation	None. Yes.	None.	None. Yes.	None.

* Half Fourier Single-shot Turbo-spin Echo sequence.

**Fast low-angle shot magnetic resonance imaging.

### Image interpretation

All images were analysed by use of Amira 5.3.2 (Visage Imaging, San Diego, USA). The programme was also used to assess the volumetric data, allowing to select any plane of view. The signal intensity values of regions of interest (ROI) in the liver and spleen were visually assessed on T1-weighted images and recorded on T2-weighted MR-images with and without fat saturation.[18] The ROIs were drawn to be around 1 cm with a circular tool. Three ROIs were obtained in the liver (one in the left lobe, two in the right lobe) in three sections and selected to include levels above, at, and below the portal vein. Regions of vessels and artefacts were avoided and the standard deviation of the signal intensity measurement within the ROIs was kept below 10%. To account for signal heterogeneity, the signal intensity of the liver was recorded as the average measurement of two readers from the nine respective ROIs placed in the liver. The signal intensity of the spleen was measured with a 1 cm ROI, placed in three different sections of the spleen. The mean signal intensity was calculated from three ROIs in different sections as an average measurement of the two readers.

The liver fat was quantified on the T2-weighted images as the relative loss of signal intensity on fat-saturated MRI images with the following formula: (SI_nonfat_—SI_fat_/SI_nonfat_) * 100, with SI being the mean liver signal intensity divided by the mean spleen signal intensity [18]. SI_nonfat_ refers to images without fat-saturation and SI_fat_ to images with fat saturation [18].

Due to the acknowledged effects of liver fat on the signal intensity of fat-saturated MR images, we considered the measurement of relative signal intensity loss to be an adequate approximation of the actual difference in the amount of liver fat in underweight and normal weight patients.

Liver fat content was graded on a 0–3 scale, whereby grade 0 (normal) referred to absence of steatosis with a measured liver fat content of less than 5%, grade 1 (mild) was associated with a liver fat content of 5–33%, grade 2 (moderate) referred to a liver fat content of 34% to 66% and grade 3 (severe) showed a liver fat content of 67% or greater [19].

### Liver and spleen size measurements

Diameters were measured in straight lines using axial and coronal MR images. They were measured in maximum extension in craniocaudal, anteroposterior and transverse distances and also as a craniocaudal distance in the mid-clavicular line [20].

To estimate the volume of the spleen, the splenic index was calculated, based on the maximum craniocaudal diameter, the maximum dimension on the axial scan and the maximum thickness on the axial scan. The upper limit for splenomegaly was set at 480 [21].

### Calculation of NAFLD and FIB-4-scores

Both the NAFLD (NFS) and the FIB-4 score are indicators of liver fibrosis. The NFS aims to differentiate between NAFLS patients without (F0-F2) and with (F3-F4) advanced liver fibrosis, selecting two cut-off points for the presence (> 0.676) and absence (< -1.455) of significant fibrosis [22]. A higher NFS was found to be a surrogate marker for the progression of liver fibrosis, being significantly predictive of death in patients with NAFLD [23]. The FIB-4 score is alternative scoring system for ruling out advanced stages of fibrosis, with a score below 1.45 having a negative predictive value of over 90% for advanced liver fibrosis and a score of over 3.25 having a positive predictive value of 65% for advanced fibrosis with a specificity of 97% [24].

**NFS score**:

$$\begin{array}{l} (-1.675+0.037\times \left(\text{age}\left[\text{years}\right]\right)+\left(0.094\times \text{BMI}\left[\text{kg}/{\text{m}}^{2}\right]\right)+\left(1.13\times \text{impaired}\;\text{fasting}\;\text{glucose}\;\text{or}\;\text{diabetes}\left[\text{yes}=1,\;\text{no}=0\right]\right)\\ +\left(0.99\times \text{AST}/\text{ALT}\;\text{ratio}\right)-\left(0.013\times \text{platelet}\left[109/\text{L}\right]\right)-\left(0.66\times \text{albumin}\left[\text{g}/\text{dl}\right]\right) \end{array}$$

[22].

**FIB-4-score**:

$$\text{Age}\left(\text{years}\right)\times \text{AST}\left(\text{U}/\text{L}\right)/[\text{PLT}(10^{9}/\text{L})\times {\text{ALT}}^{1/2}\left(\text{U}/\text{L}\right)].$$

### Statistical analysis

Statistical analysis was performed with “R” Statistical Software (Version 3.4.0. R Development Core Team. 2017). Data were expressed as means ± standard deviations. The distribution of continuous variables, such as years of disease or laboratory values, were visualized by violin plots. Student t-tests were used for continuous variables and chi-squared (χ^2^) test was used for dichotomous variables. Univariable and descriptive analyses were performed to assess the distribution of study variables and the relationship between dependent and independent variables. Regression models were used to assess if underweight as the tested influencing factor for NAFLD (age-, gender- and disease-matched controls) was potentially confounded by covariates such as prior or present medication or history of surgery. Therefore, the regression model for each covariate used the dichotomized covariate as a target and the identification of patient groups (cases/controls) as an influencing factor. In a sub-analysis, a regression model, adjusted for age, gender and field strength, was used to assess if the degree of steatosis was higher in patients with severe underweight (<17.5). If necessary, logarithmic transformation was used to reduce skewness prior to regression analysis. Interobserver reproducibility and possible differences were analyzed using linear regression analyses. In addition, Bland-Altman plots with prediction intervals were computed for the measured data to display the distribution of measurements as well as the limits of agreement. A p-value of less than 0.05 was considered statistically significant.

## Results

### Study population

A total of 61 age-, gender- and disease-matched underweight patients and 61 normal weight controls received abdominal MRI with fat saturated T2-weighted sequences at our institution (25 patients at 1.5 T, 36 patients at 3T) and were used for analysis. Out of these, 47 patients suffered from Crohn’s disease, 6 patients had a diagnosis of ulcerative colitis and 8 patients had symptoms of abdominal pain, but no physical diagnosis. Characteristics of study patients are shown in Table 2. 29 patients were severely underweight (n = 10 for 1.5 T, n = 19 for 3T). The mean age was 30.9 ± 8.9 and there were 41 females (67.2%) and 20 males (32.8%).

**Table 2:** Characteristics of the study population.

	Underweight (cases)	Normal weight (controls)
Number of cases (controls)	65	65
Number of women/men (percentage)	45/20 (69.2%/30.8%)	45/20 (69.2%/30.8%)
Mean age (range; SD)	30.9 (18–49; 8.9)	30.8 (18–49; 8.7)
Mean BMI (range; SD)	17.0 (10.7–18.4)	22.6 (20–24.9)
Cases with severe underweight (<17.5) (number (percentage))	32 (49.2%)	None.
Age at IBD onset	25.8 ± 6.9	26.3 ± 6.2
Crohn’s disease (number. percentage)	47 (72.3%)	47 (72.3%)
Ulcerative colitis (number. percentage)	6 (9.2%)	6 (9.2%)
No diagnosis (number. percentage)	12 (18.5%)	12 (18.5%)
Number (percentage) of steatosis ≥5%)	57 (87.6%)	14 (21.5%)
Number (percentage) of steatosis (>33%)	12 (18.5%)	1 (1.5%)
Number (percentage) of patients without steatosis (<5%)	8 (12.3%)	50 (76.9%)

### Comparison of characteristics of underweight and normal weight patients

**Fig 1:**
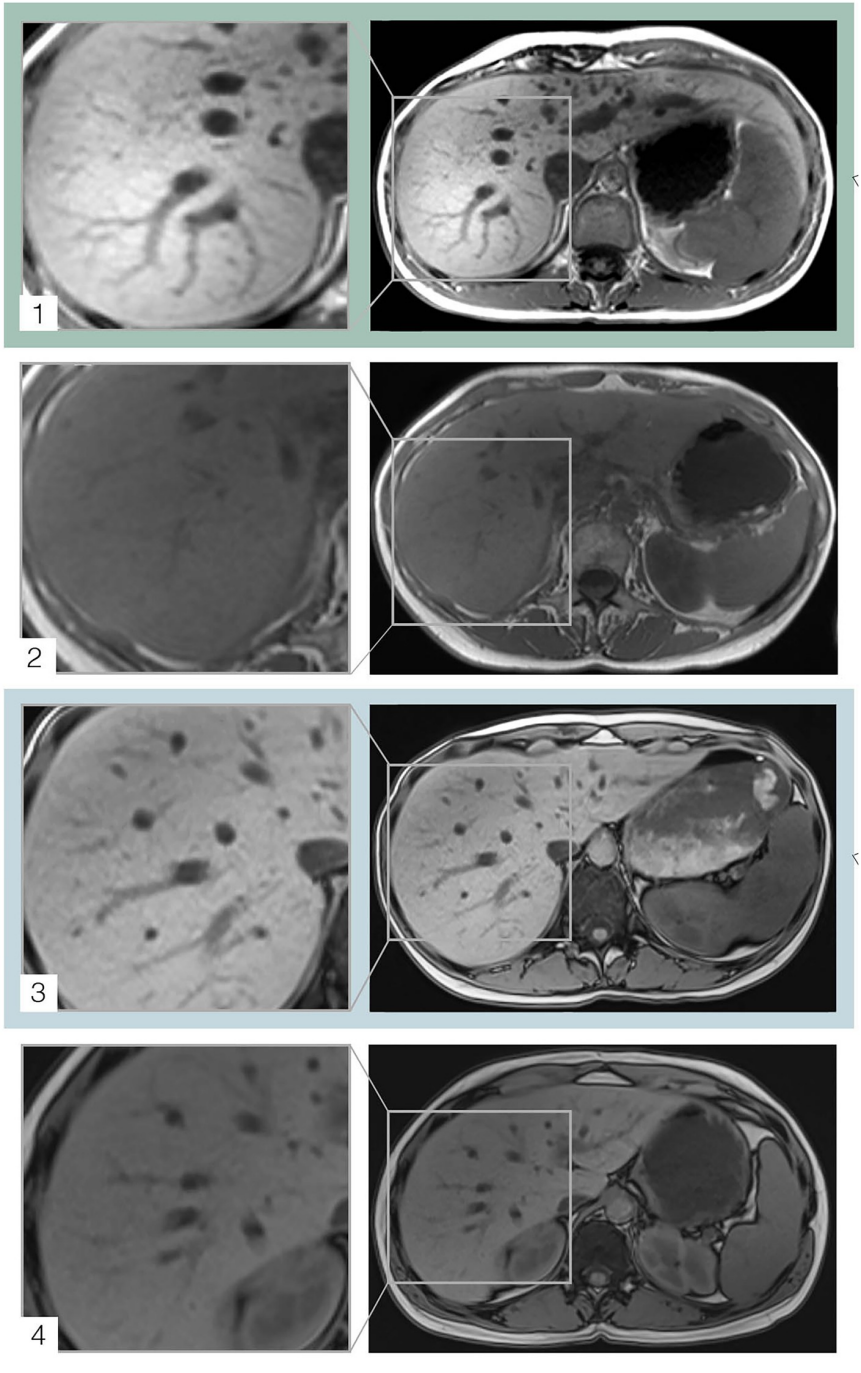
Patient case examples. The T1-weighted image 1 (with magnification on the left) shows a patient case example of a 21-year old woman with indistinct stomach pains, but without diagnosis of physical disorders, who was examined at 3T. The patient was revealed to have a fatty liver with hyperintensity on the T1-weighted sequence. The T1 weighted image 2 (with magnification on the left) shows the corresponding normal weight control (22 years, female, no diagnosis), who was also examined at a field strength of 3T, but who does not show any sign of fatty liver and no hyperintensity on the T1-weighted image. The T1-weighted image 3 (with magnification on the left) shows a patient case example of a 27-year old woman with Crohn’s Disease, who was examined at 1.5 T. The patient was revealed to have a fatty liver with hyperintensity on the T1-weighted sequence. The T1 weighted image 4 (with magnification on the left) shows the corresponding normal weight control (29 years, female, Crohn’s disease), who was also examined at a field strength of 1.5 T, but who does not show any sign of fatty liver with a normal liver signal on the T1-weighted image. The left part of the figure shows the corresponding T2 images without (1a, 3a) and with (1b, 3b) fat saturation, revealing a relative signal loss of 36% (1b) or 23% (1b).

The bottom part of Table 2 summarizes the prevalence of NAFLD/steatosis in the underweight cases and the normal weight age-, gender and diseased-matched controls. The prevalence of NAFLD/steatosis, which is defined as a measured intrahepatic fat content of at least 5%, is shown to be significantly higher in underweight patients compared to normal weight patients (88.5% versus 23%, p < 0.001). While 11 underweight patients (18%) showed moderate steatosis with a liver fat content of more than 33%, there was only 1 (1.6%) normal weight control with a liver fat content of more than 25%. None of the patients were associated with severe steatosis and a fat liver content above 66%. See Fig 1 for with visualization of steatosis in underweight study patients compared to normal weight controls. It presents case examples of moderate and mild steatosis in a severely underweight patient compared to normal weight controls, examined with T1 weighted images at 3T and 1.5T, providing the corresponding T2 weighted images with and without fat saturation for the two underweight patients with steatosis.

### Evaluation of differences of T2-based liver fat quantification

**Fig 2:**
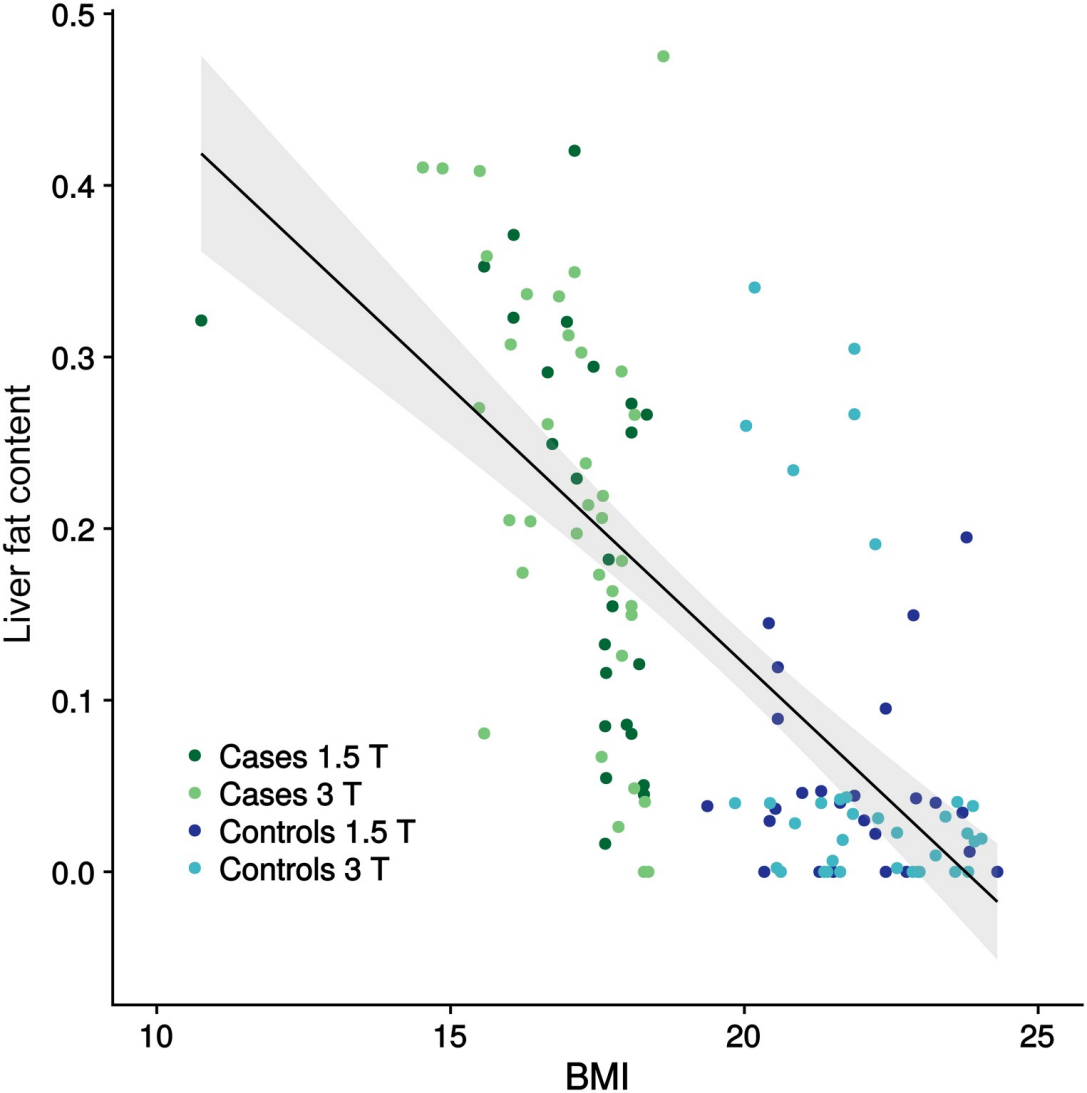
Association between apparent liver fat content and BMI. Association between apparent liver fat content and BMI for cases and controls, displayed by a scatter plot with linear regression line and corresponding 95% confidence interval (adjacent grey area). Cases are represented by green dots (dark green for 1.5T, light green for 3T), while controls are indicated by blue dots (dark blue for 1.5T, light blue for 3T). The liver fat content appears to be higher in patients with underweight BMI range than in patients with normal BMI range.

Lower BMI values in underweight patients were significantly associated with higher liver fat content (refer to Fig 2). Patients from the underweight study population showed significantly higher liver fat percentages. By comparison, the liver fat content of the controls was reduced by -0.18 units on average (18%) (95% Cl: -0.21; -0.14). There were no significant differences between the observers or at different field strengths (p > 0.05).

**Fig 3:**
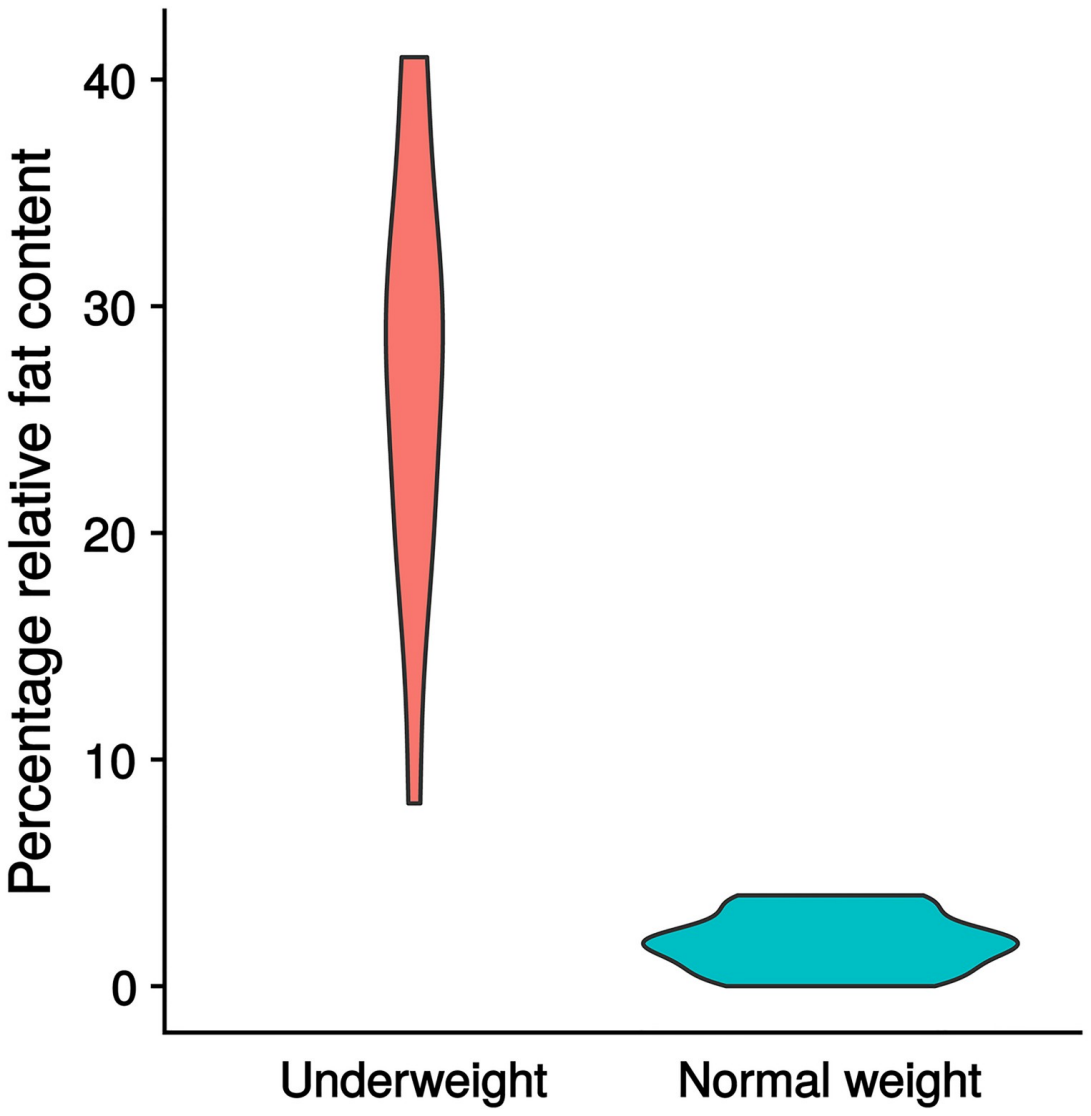
Differences in percentage relative liver fat content between underweight individuals and normal weight controls. This figure refers to a subgroup of underweight individuals (n = 12) without inflammatory bowel disease and visualizes the differences in percentage relative liver fat content between cases and controls and also between the observers. It can be seen, that there is a clear difference between cases and controls, with cases showing a significantly higher percentage liver fat content. There are no significant differences between the two observers.

If the analysis of liver fat content was repeated for the small subgroup of underweight individuals without IBD (n = 8), similar results were obtained. Compared to the underweight cases, the liver fat content of the controls was reduced by -0.23 units on average (23%) (95% Cl: -0.33; -0.13]). Consequently, underweight patients without diagnosis showed significantly higher liver fat percentages (p < 0.05). Refer to Fig 3 for visualization of the differences in percentage relative liver fat content between cases and controls.

### Sub-analysis for patients with extremely low body weight

Patients with extremely low body weight (BMI <17.5 kg/m^2^, n = 29) showed significantly increased liver fat percentages compared to underweight patients with a BMI of 15-5-18.5 kg/m^2^ (p < 0.05). A linear regression model, which was adjusted for age, gender and field strength, demonstrated that the liver fat content of patients with extremely low body weight was increased by 0.14 units (14%) on average (95% CI: 0.08; 0.19).

### Comparison of medical treatments and surgical therapies

To investigate whether the type of medical treatments and surgical therapies would differ between the case group and the control group, a logistic regression model was used. There were no indications of significant differences in the type of medications between cases and controls (all p-values > 0.05). With regard to surgery, underweight cases had prior colon resections more frequently compared to normal weight controls (22.0% versus 10.2%, refer to Table 3). However, the difference was not significant (χ^2^ = 7.97, p = 0.08), which was confirmed by the multinomial logistic regression model applied. In addition, disease duration, (steroid) medication, extent of bowel surgery and c-reactive protein (CRP) showed no significant association with hepatic steatosis for both cases and controls (p > 0.05).

**Table 3:** Disease-related medical and surgical therapy.

	Underweight (cases)	Normal weight (controls)
Any bowel surgery (percentage)	26 (40%, 4 missings)	23 (35.4%, 2 missings)
Terminal ileum resection (percentage)	13 (20%)	15 (24.6%)
Colon resections (percentage)	13 (20%)	6 (9.2%)
Sigmoid colectomy (percentage)	0	2 (3.1%)
Use of steroids	18	17
Use of immunomodulators (Azathioprine. Methotrexate)	28	34
Use of Mesalazine	14	7
Use of TNF-alpha biologics*	35	31

* TNF = Tumor necrosis factor

### Comparison of years of disease

The mean difference in the distribution of disease years for cases and controls was 0.39 years (95% confidence interval (CI): -3.22; 2.44). If it was additionally adjusted for age and gender using a linear model, there was a mean difference of 0.2 years (95% uncertainty interval:-2.28; 2.69). Among patients with steatosis/NAFLD the mean age at the time of diagnosis of IBD was 25.7 ± 6.8 years compared to 26.1 ± 6.0 years for the controls. As a consequence, no significant difference could be observed with regard to the mean years of disease between the cases and controls (p > 0.05).

### Comparison of laboratory values

**Fig 4:**
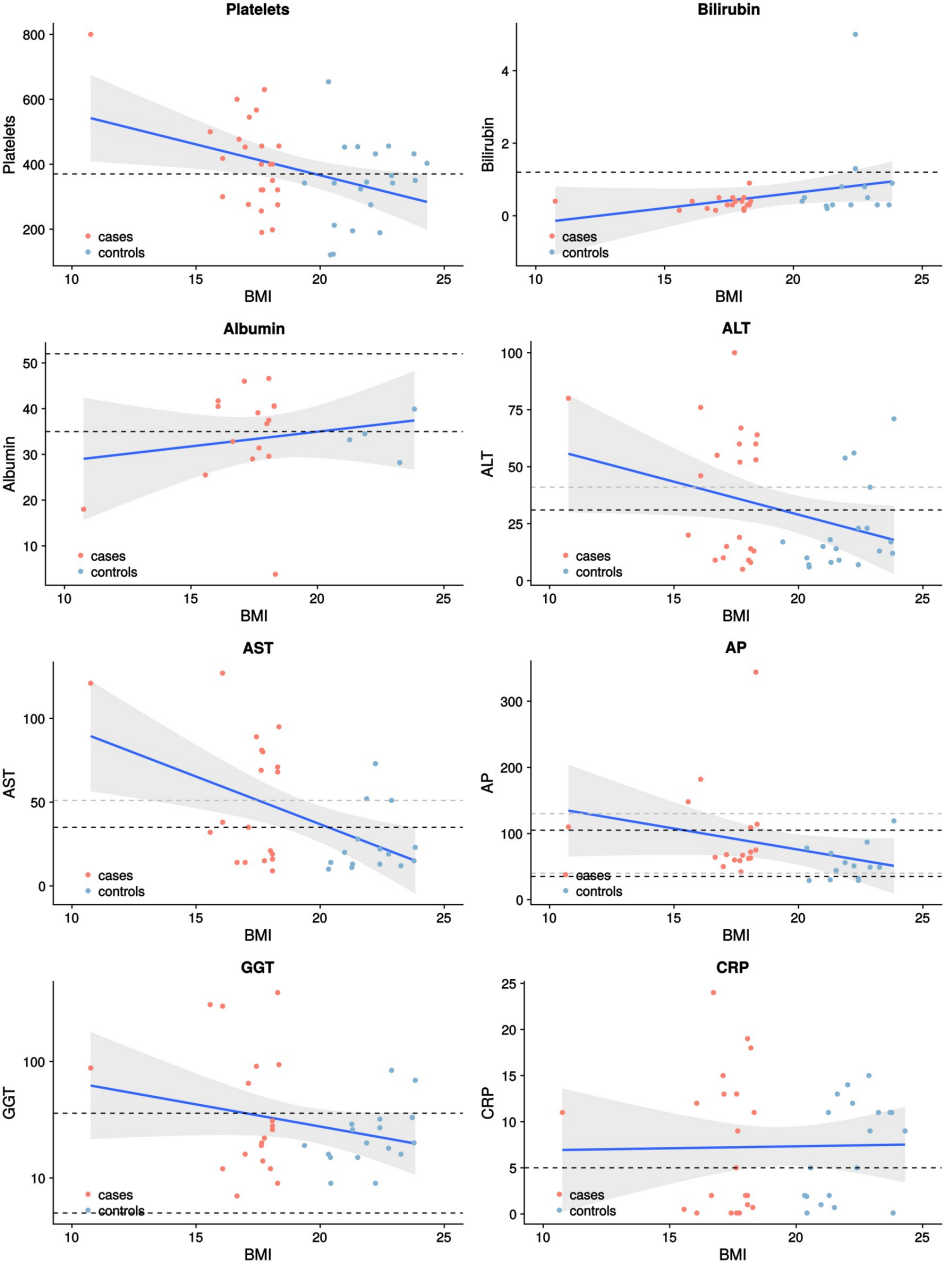
Laboratory values and BMI. Association between various laboratory values and BMI for cases and controls. Cases are represented by red dots, while controls are indicated by blue dots. The blue graphs correspond to linear regression lines, while the adjacent grey areas represent the 95% confidence intervals. The limits for the laboratory values are as follows: Albumin (Alb): 35–52 g/L; alanine aminotransferase (ALT): women—< 31 U/L, men—< 41 U/L; alkaline phosphatase (AP): women– 35–105 U/L, men– 40–130 U/L; aspartate aminotransferase (AST): women < 35 U/L, men <50 U/L; total bilirubin (Bilirubin) < 1.2 mg/dl and gamma-glutamyltransferase (GGT): women– 5–36 U/L, men– 8–61 U/L.; CRP < 5 mg/L. In the graphs the limits for women are marked by the black dotted lines, while the limits for men are marked by grey dotted lines. For laboratory values without sex-specific values, only black dotted lines are used.

**Fig 5:**
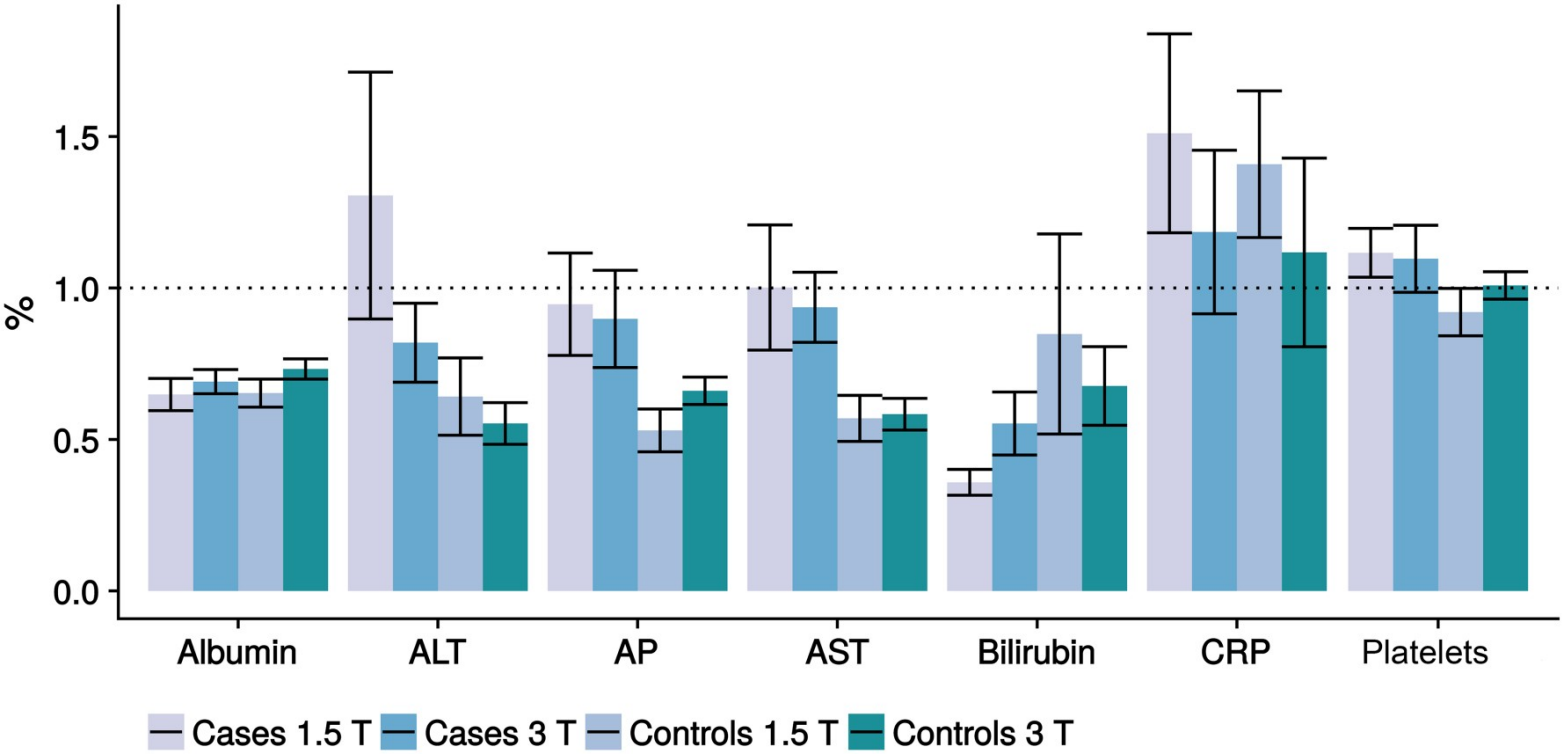
Distribution of laboratory values. Laboratory values as mean percentages of the upper limit of normal with the corresponding standard deviations, including Albumin, ALT (Alanin transaminase), AP (Alkaline phosphatase), AST (Aspartate aminotransferase), Bilirubin, CRP (C-reactive protein) and platelets. Especially CRP, ALT and platelets show increases above 100% of the upper limit of normal. For ALT, AST and AP, there were significant differences between cases and controls (p<0.05), with each case showing higher laboratory values compared to controls, although most of them remained within normal limits.

Fig 4 displays the distribution of laboratory values between the cases and controls. Both the t-test and the linear model measured significant differences in the distributions of AST, AP and GGT, with the cases showing increased average values (refer to Table 4). For the normal weight controls, the risk of finding laboratory parameters above the upper limit is significantly reduced (interval of odds ratios below one), while the risk of observing laboratory values above the limit is significantly increased for the underweight study patients. The logistic regression analysis also recognizes a similar correlation for the laboratory value ALT, with a higher number of patients with NAFLD showing an elevated ALT (25.5% vs. 10.2%; p ≤ 0.05). Fig 5 displays laboratory values as mean percentages of the upper limit of normal with the corresponding standard deviations.

**Table 4:** Analysis of potential differences between case/control group regarding laboratory values.

Laboratory value	cases (n) below	cases (n) above	controls (n) below	controls (n) above	p-value cases/controls
Bilirubin (mg/dl)	0	2	0	7	p>0.05
Albumin (g/L)	15	1	11	0	p>0.05
ALT* (U/L)	0	14	0	5	p<0.05
AST** (U/L)	0	16	0	2	p<0.001
AP*** (U/L)	1	14	5	3	p<0.05
GGT****(U/L)	0	15	1	3	p>0.05
CRP***** (mg/L)	0	11	0	10	p>0.05

*Alanin transaminase,

**Aspartate aminotransferase,

***Alkaline phosphatase,

****GGT Gamma-glutamyl transferase,

***** C-reactive protein.

Analysis of the differences in laboratory values between cases and controls. The first four columns of this table show the number of observations below or above the specified limits, divided by case and control group. The last column shows the corresponding p-values, when testing for significant differences between cases and controls.

### Comparison of liver diameter measurements

Regarding liver size based on diameter measurements, the underweight study population showed a midclavicular line average of 14.8 ± 2.6 cm compared to a midclavicular line average of 13.8 ± 2.5 cm in the cases (p < 0.05). The maximal anteroposterior diameter in the transversal plane was 15.7 ± 1.6 cm for the cases compared to 14.8 ± 2.4 cm for the controls (p < 0.05). The maximum craniocaudal and transversal diameters were also slightly higher for the cases compared to the controls (maximum craniocaudal: 20.4 ± 2.6 cm vs. 19.5 ± 2.3 cm, transversal: 14.8 ± 2.3 cm vs. 15.7 ± 1.6 cm), but these differences did not reach significance level (p > 0.05). Taking diameter measurements as an estimate for liver size, it can be assumed that study patients showed marginally enlarged livers at most, however, with higher diameters compared to controls.

### Comparison of spleen size measurements

For 1.5T, the splenic index was 501.3 ± 205.5 for cases and 409 ± 134.2 for controls and for 3T, the splenic index was 491.3 ± 216.3 for cases and 398.0 ± 111.1 for controls. For both 1.5 T and 3T, no significant differences in spleen size could be observed between cases and controls, although the spleen index in the case groups tended to be higher with a trend for statistical significance (1.5 T: p = 0.06; 3 T: p = 0.12).

### Assessment of NAFLD and FIB-4-scores

While the NFS and the FIB-4 score were slightly higher for the cases compared to the controls (NFS—1.5T: -24.8 versus -26.6; 3T: -26.9 versus -27.6) (FIB-4–1.5T: 0.043 versus 0.039; 3T: 0.040 versus 0.037, these differences did not reach significance. Furthermore, based on the scores we found no cases suspicious of advanced liver fibrosis. This is in accordance with our MRI results, where we observed multiple cases of mild to moderate steatosis in underweight (IBD) patients, but could not find any signs of significant fibrosis or cirrhosis.

### Interobserver agreement

**Fig 6:**
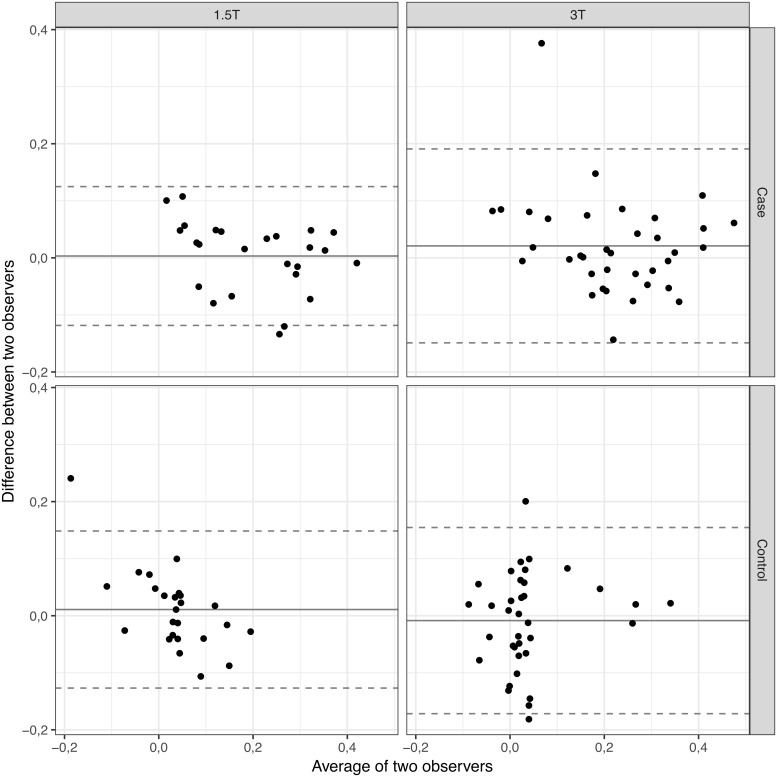
Interobserver variability. Bland-Altman plots for the assessment of interobserver variability for percentage relative signal loss on the fat saturated T2 weighted images (percentage liver fat content) between cases and controls and at different field strengths. The mean ratio of the data is graphed with the centre line. Upper and lower reference lines show the upper and lower limits of agreement (95% prediction intervals).

The visual illustration of interobserver agreement for the measurement of the liver fat percentages, adjusted by field strengths as well as cases/controls, is provided by the Bland-Altman-plots in Fig 6, displaying the distribution of measurements and the limits of agreement.

## Discussion

This case-control study assessed the frequency of NAFLD in patients with significantly low body weight compared to patients with normal weight. Underweight patients demonstrated significantly higher liver fat percentages compared to the normal weight patients, corresponding to mild to moderate liver steatosis. Also, underweight patients showed slightly increased liver enzymes and liver diameters, hinting at initial metabolic disturbances.

NAFLD is a global public health problem and one of the most common causes of chronic liver disease. Although simple fatty liver is a benign condition, 10–20% can progress to non-alcoholic steatohepatitis (NASH), which dramatically increases the risk for cirrhosis and end-stage liver disease [25]. Currently, NAFLD is widely considered a manifestation of the metabolic syndrome in close relation to obesity. In patients with IBD, NAFLD was previously reported in approximately 10% of the cases [26], whereby, apart from medication and bowel surgery, it was similarly linked to increasing metabolic risk factors and overweight. However, NAFLD was also found to occur in lean as well as severely underweight subjects [7, 27].

To our knowledge, in the context of IBD, the presence of NAFLD has not been examined in underweight patients so far. It is possible, that it may frequently be unnoticed in this collective. Previous studies on NAFLD might have underestimated the prevalence of mild to moderate hepatic steatosis in lean to underweight patients due to most of them being ultrasound-based with poor accuracy for hepatic steatosis < 30% [28]. Especially mild steatosis might constitute an important proportion among underweight patients with IBD.

Although the phenotype of IBD is changing with the prevalence of obesity and overweight patients gradually rising [29], approximately one in six patients still suffer from significant malnutrition, often related to more severe forms of disease and accompanied by self-imposed food restriction behaviour [30]. Apart from the acknowledged influence of medication and bowel surgery, presence of NAFLD in underweight IBD patients could, furthermore, be an indicator of and independent and different pathophysiology, which is not associated with obesity or insulin resistance [31, 32].

While compared to the general population, IBD patients usually have a lower BMI and a lower prevalence of metabolic risk factors [14], within IBD populations, IBD patients with NAFLD have been found to be older, more often with a higher average BMI and more often diabetics [32]. Taking into account the severity of IBD, Sartini et al. suggested the prevalence of different IBD phenotype, with less aggressive forms of IBD being associated with mild-to-moderate steatosis, and more severe forms of IBD with frequent relapses being related to severe steatosis at ultrasound [31]. As in the current study, regarding IBD activity assessment, there were no data available on endoscopic severity or clinical activity indices, it cannot be excluded, that lower weight IBD patients suffered from higher disease activity compared to normal weight IBD patients, even though showing a similar distribution of disease duration, (steroid) medication and bowel surgery [33]. But to this end, our results would be in contrast to Sartini et al., as in the present study, underweight patients with possibly higher disease activity had mild-to-moderate instead of severe steatosis. With a subgroup of twelve underweight patients without IBD showing a 25% higher liver fat content compared to the corresponding normal weight non-IBD controls, the results of the present study may hint at a synergistic effect of IBD and malnutrition in the pathogenesis of IBD, with underweight NAFLD representing a distinct entity. However, larger numbers of underweight non-IBD patients with normal weight controls are needed to support this hypothesis.

To date, evidence for the relationship between underweight and NAFLD remains scarce. A previous study, developing a malnutrition animal model, suggested that severe malnutrition might lead to an impaired function of liver mitochondria and a loss of peroxisomes, which are important to maintain the normal liver function [7]. Another example is the widely-used methionine and choline-deficient diet (MCD) mouse model, in which mice submitted to the MCD diet lose weight, but show fat accumulation in the liver due to beta-oxidation of fatty acids and very low density lipoproteins (VLDL) with resulting triglycerides export from the cell [34, 35]. Furthermore, in some cases NAFLD can also by driven by single-nucleotide polymorphisms, such as in the gene for phospholipase A3 (PNPLA43). PNPLA43 was strongly associated with increased triglycerides in the hepatic content due to increased lipogenesis, but at the same time shown to be independent of overweight or diabetes, so that it might not have any influence on metabolic syndrome components and may also be present in lean individuals [36]. A recent longitudinal study by Hagström et al. found that lean patients with NAFLD, while showing lower stages of fibrosis and not having an increased risk of overall mortality, were at a higher risk of severe liver disease, independent of available confounders [37]. This highlights the fact that NAFLD in lean or underweight patients is not a simple benign condition. Studies focusing on severely malnourished patients with anorexia found elevated liver enzymes as indicating NAFLD to be common, especially in patients with very low body weight (BMI < 12 kg/m^2^) [6, 38, 39]. Anorexia-induced lipolysis was discovered to promote late triglyceride and free fatty acid accumulation in the liver and kidney [40]. A previous study suggested an increase in intrahepatic lipid content, following 36 hours of fasting, with a direct association to plasma levels of 3-hydroxybutyrate, which might also serve as an explanation for exacerbations of NAFLD with steatohepatitis seen in patients with anorexia nervosa [41].

At our institution, we observed that patients with significant underweight often showed fatty liver on MRI scans, which was confirmed by the results of the present analysis. Comparing the cases and controls, no significant difference could be found with regard to the general use of medical therapies, especially including potentially hepatotoxic medications such as corticosteroids, immunomodulators or 5-amino-salicylic acids. Regarding a possible association between IBD disease activity and fatty liver, Carr et al. previously reported an absent association between IBD severity and degree of NAFLD [42]. Even though in the present study IBD disease activity was not assessed with regard to endoscopic severity or clinical activity indices, activity-related parameters such as disease duration, (steroid) medication, extent of bowel surgery and c-reactive protein (CRP) showed no significant association with hepatic steatosis for both cases and controls. While prior studies on NAFLD in IBD patients reported mean ages of approximately 45 years [15, 43, 44], the present population has a strikingly lower average age of approximately 30 years. Initial steatosis in young underweight IBD patients may thus have been underestimated previously.

NAFLD includes different forms of liver disease, such as simple steatosis, non-alcoholic steatohepatitis (NASH), liver fibrosis, cirrhosis, or even hepatocellular carcinoma [45]. The transition from steatosis to steatohepatitis, so far, remains insufficiently enlightened. The role played by the gut in NAFLD is still controversial, even though animal and human studies supported the relevance of a gut derived endotoxin [46]. In the gut-liver-axis the main players are the microbiota of the gut, gut-derived bacterial components and the intestinal barrier. Changes in microbiology and intestinal permeability can promote the translocation of bacterial components into the portal vein, the activation of inflammation by signalling in hepatocytes and finally the transition from simple steatosis to NASH [47]. A previous study by Miele et al. found intestinal permeability to be increased in patients with NAFLD and to correlate with severity of steatosis, suggesting it to be important in the pathogenesis of hepatic fat deposition [48]. However, further advances in the understanding of the gut-liver-axis are warranted, as this may improve management of liver diseases in the future. Currently, it remains questionable, if NASH will be treated as an infectious disease [49].

In our study population, there was no suspicion of NASH in any of the patients. Regarding fibrosis serum markers, AST, ALT, platelet count, GGT, bilirubin and albumin were available for the majority of patients. However, non-invasive tests for liver fibrosis, such as the NAFLD score or FIB-4-score did not identify any cases suspicious of advanced liver fibrosis. As NASH was previously described to be more common and potentially more progressive in the setting of diabetes mellitus, overweight and older age[50], a possible explanation for the apparent absence of NASH in the present NAFLD population could be the lack of diabetes disease, lower weight and young age. But even low-to-moderate steatosis without signs of NASH, as was found in the present study, carries a clinical burden and was previously linked to severe liver disease and cardiovascular risk [51].

As NAFLD is mostly silent, it is often discovered accidentally through clinical examination in form of hepatomegaly, imaging or elevated liver enzymes at a later stage. In the clinical setting, NAFLD is usually diagnosed via abdominal ultrasound (US), as liver biopsies are not practically feasible for assessing NAFLD, while they are essential for diagnosis of NASH and differentiation of NAFLD from NASH, despite being limited by sampling variability [52]. Previous research on NAFLD included liver biopsy data, which are recognized as the key test for chronic liver disease, but, given their invasiveness, carry the risk for procedural complications, such as bleedings [53]. One study included 108 NAFLD patients with serial liver biopsies more than a year apart, finding progression of steatosis to NASH in 44% of the patients with baseline NAFLD [54]. Dela Cruz et al. presented more than 1,000 patients with biopsy-confirmed NAFLD and BMI values < 25 kg/m^2^, finding NAFLD patients with normal BMI to have a higher mortality compared to NAFLD patients with overweight [55]. There are far less biopsies in the literature for IBD patients. McGowan et al. recently reported a series of seven patients with Crohn’s Disease and biopsy proven NAFLD [14] and Sourianarayanane et al. reported six NAFLD patients with liver biopsies. [15] A prospective analysis of 200 ulcerative colitis patients found biopsy-proven NAFLD in 11.2% of the patients [56].

While US, due to its cost-effectiveness, is the most frequently used imaging tool for diagnosis of NAFLD, it has a low accuracy for detecting mild steatosis [57]. Magnetic resonance imaging (MRI), by contrast, is regarded as the most accurate practical method for measuring fatty liver in clinical practice and—other than US and computed tomography (CT), which evaluate hepatic steatosis through parameters such as echogenicity and attenuation, enables direct measurement of the quantity of hepatic fat [57]. At this, MRI achieves sensitivities and specificities of 76.7–91% and 80–87% in detecting histologic steatosis ≥ 5%.[58, 59] Also, MRI is the modality of choice for non-invasive quantification of steatosis. Qayyum et al. previously developed a simple method for liver fat quantification, based on T2 weighted imaging with fat saturation, and compared it to out-of-phase-gradient-echo-imaging, indicating that it was more robust for fat quantification [18]. Therefore, we made use of this proposed method in the present study. The fact that the measured liver fat quantity did not significantly differ between 1.5 T and 3 T supports the clinical applicability of this approach.

With regard to clinical practice, we would recommend not only the monitoring of conventional NAFLD risk factors, such as metabolic syndrome, diabetes or BMI/waist circumference, but also the non-invasive monitoring of liver steatosis in significantly underweight (IBD) patients. Especially patients with IBD, who often undergo frequent MRI in clinical routine, could benefit from the additional evaluation of MRI scans with regard to steatosis to recognize early changes in the liver tissue.

Further investigations are needed in NAFLD patients to identify possible factors other than metabolic pathogenic IBD-related causes and also, specifically in a set of underweight patients without diagnosis of physical disorders, to better understand the association between relevant underweight and liver disease.

The present study has several limitations, which need to be acknowledged in the interpretation of the outcomes: Firstly, this cross-sectional case-control study is retrospective. Secondly, diagnosis of NAFLD was based on MRI and no liver biopsy was performed. However, due to its invasiveness and the possibility of false negative results due to sampling a relatively small area, liver biopsies are not practically feasible for diagnosis of NAFLD in general [60]. As a consequence, MRI is regarded as the most accurate practical method for measuring fatty liver in clinical practice, with a higher diagnostic performance compared to US and CT. However, fat suppression may be imperfect, e.g. due to inhomogeneities in the magnetic field. Furthermore, spectral complexity of fat and T2 decay may result in discrepancies between the true fat fraction and the apparent fraction, measured with MRI. Another aspect is, that two different field strengths (1.5T/3T) were used. However, we could not find any significant differences regarding the results of liver fat quantification with similar liver fat content measurements for 1.5T and 3T. The reproducibility of MR-based liver fat quantification across different field strength is also supported by previous research [61, 62]. Furthermore, due to the assessment of alcohol consumption relying on prior clinical evaluation in the medical records, it cannot be ruled out that individual patients may have exceeded cut-off limits for alcohol consumption (20 g/d for women and 30 g/d for men) [63]. Finally, the effect of weight gain on NAFLD could not be considered, which would have been interesting, as a previous study suggested, that in patients suffering from anorexia with severe malnutrition, steatosis especially occurred in the process of refeeding [39].

### Conclusions

Underweight patients showed a higher risk for mild to moderate NAFLD compared to the matched controls with normal weight. Presence of fatty liver in underweight patients should not be neglected, as it could indicate a higher risk for metabolic disorders or even hint at a different entity of non-metabolic NAFLD. Further investigations, specifically in a set of underweight patients without diagnosis of physical disorders are needed to better understand the association between relevant underweight and liver disease.

## References

[1] Byrne CD, Targher G. NAFLD: a multisystem disease. J Hepatol. 2015;62(1 Suppl):S47–64. Epub 2015/04/29. 10.1016/j.jhep.2014.12.012 . 25920090 10.1016/j.jhep.2014.12.012

[2] Le MH, Devaki P, Ha NB, Jun DW, Te HS, Cheung RC, et al Prevalence of non-alcoholic fatty liver disease and risk factors for advanced fibrosis and mortality in the United States. PLoS One. 2017;12(3):e0173499 Epub 2017/03/28. 10.1371/journal.pone.0173499 28346543 10.1371/journal.pone.0173499 PMC5367688

[3] Cortez-Pinto H, Camilo ME, Baptista A, De Oliveira AG, De Moura MC. Non-alcoholic fatty liver: another feature of the metabolic syndrome? Clin Nutr. 1999;18(6):353–8. Epub 2000/01/15. . 10634920 10.1016/s0261-5614(99)80015-6

[4] Das K, Das K, Mukherjee PS, Ghosh A, Ghosh S, Mridha AR, et al Nonobese population in a developing country has a high prevalence of nonalcoholic fatty liver and significant liver disease. Hepatology. 2010;51(5):1593–602. Epub 2010/03/12. 10.1002/hep.23567 . 20222092 10.1002/hep.23567

[5] Rautou PE, Cazals-Hatem D, Moreau R, Francoz C, Feldmann G, Lebrec D, et al Acute liver cell damage in patients with anorexia nervosa: a possible role of starvation-induced hepatocyte autophagy. Gastroenterology. 2008;135(3):840–8, 8.e1–3. Epub 2008/07/23. 10.1053/j.gastro.2008.05.055 . 18644371 10.1053/j.gastro.2008.05.055

[6] Rosen E, Bakshi N, Watters A, Rosen HR, Mehler PS. Hepatic Complications of Anorexia Nervosa. Dig Dis Sci. 2017;62(11):2977–81. Epub 2017/09/22. 10.1007/s10620-017-4766-9 . 28932925 10.1007/s10620-017-4766-9

[7] van Zutphen T, Ciapaite J, Bloks VW, Ackereley C, Gerding A, Jurdzinski A, et al Malnutrition-associated liver steatosis and ATP depletion is caused by peroxisomal and mitochondrial dysfunction. J Hepatol. 2016;65(6):1198–208. Epub 2016/06/18. 10.1016/j.jhep.2016.05.046 . 27312946 10.1016/j.jhep.2016.05.046

[8] Garre MA, Boles JM, Youinou PY. Current concepts in immune derangement due to undernutrition. JPEN J Parenter Enteral Nutr. 1987;11(3):309–13. Epub 1987/05/01. 10.1177/0148607187011003309 . 2955137 10.1177/0148607187011003309

[9] Cederholm T, Bosaeus I, Barazzoni R, Bauer J, Van Gossum A, Klek S, et al Diagnostic criteria for malnutrition—An ESPEN Consensus Statement. Clin Nutr. 2015;34(3):335–40. Epub 2015/03/24. 10.1016/j.clnu.2015.03.001 . 25799486 10.1016/j.clnu.2015.03.001

[10] Fisher RL. Wasting in chronic gastrointestinal diseases. J Nutr. 1999;129(1S Suppl):252S–5S. Epub 1999/01/23. 10.1093/jn/129.1.252S . 9915909 10.1093/jn/129.1.252S

[11] Rocha R, Santana GO, Almeida N, Lyra AC. Analysis of fat and muscle mass in patients with inflammatory bowel disease during remission and active phase. Br J Nutr. 2009;101(5):676–9. Epub 2008/07/18. 10.1017/S0007114508032224 . 18631418 10.1017/S0007114508032224

[12] Mijac DD, Jankovic GL, Jorga J, Krstic MN. Nutritional status in patients with active inflammatory bowel disease: prevalence of malnutrition and methods for routine nutritional assessment. Eur J Intern Med. 2010;21(4):315–9. Epub 2010/07/07. 10.1016/j.ejim.2010.04.012 . 20603043 10.1016/j.ejim.2010.04.012

[13] Bargiggia S, Maconi G, Elli M, Molteni P, Ardizzone S, Parente F, et al Sonographic prevalence of liver steatosis and biliary tract stones in patients with inflammatory bowel disease: study of 511 subjects at a single center. J Clin Gastroenterol. 2003;36(5):417–20. Epub 2003/04/19. . 12702985 10.1097/00004836-200305000-00012

[14] McGowan CE, Jones P, Long MD, Barritt ASt. Changing shape of disease: nonalcoholic fatty liver disease in Crohn’s disease-a case series and review of the literature. Inflamm Bowel Dis. 2012;18(1):49–54. Epub 2011/02/26. 10.1002/ibd.21669 21351214 10.1002/ibd.21669 PMC3137748

[15] Sourianarayanane A, Garg G, Smith TH, Butt MI, McCullough AJ, Shen B. Risk factors of non-alcoholic fatty liver disease in patients with inflammatory bowel disease. J Crohns Colitis. 2013;7(8):e279–85. Epub 2012/11/20. 10.1016/j.crohns.2012.10.015 . 23158500 10.1016/j.crohns.2012.10.015

[16] Chalasani N, Younossi Z, Lavine JE, Diehl AM, Brunt EM, Cusi K, et al The diagnosis and management of non-alcoholic fatty liver disease: practice Guideline by the American Association for the Study of Liver Diseases, American College of Gastroenterology, and the American Gastroenterological Association. Hepatology. 2012;55(6):2005–23. Epub 2012/04/11. 10.1002/hep.25762 . 22488764 10.1002/hep.25762

[17] Reilly NR, Lebwohl B, Hultcrantz R, Green PH, Ludvigsson JF. Increased risk of non-alcoholic fatty liver disease after diagnosis of celiac disease. J Hepatol. 2015;62(6):1405–11. Epub 2015/01/27. 10.1016/j.jhep.2015.01.013 25617505 10.1016/j.jhep.2015.01.013 PMC4439270

[18] Qayyum A, Goh JS, Kakar S, Yeh BM, Merriman RB, Coakley FV. Accuracy of liver fat quantification at MR imaging: comparison of out-of-phase gradient-echo and fat-saturated fast spin-echo techniques—initial experience. Radiology. 2005;237(2):507–11. Epub 2005/10/26. 10.1148/radiol.2372040539 . 16244259 10.1148/radiol.2372040539

[19] Brunt EM, Janney CG, Di Bisceglie AM, Neuschwander-Tetri BA, Bacon BR. Nonalcoholic steatohepatitis: a proposal for grading and staging the histological lesions. Am J Gastroenterol. 1999;94(9):2467–74. Epub 1999/09/14. 10.1111/j.1572-0241.1999.01377.x . 10484010 10.1111/j.1572-0241.1999.01377.x

[20] Roloff AM, Heiss P, Schneider TP, Quadrat A, Kromrey ML, Zeman F, et al Accuracy of simple approaches to assessing liver volume in radiological imaging. Abdom Radiol (NY). 2016;41(7):1293–9. Epub 2016/02/26. 10.1007/s00261-016-0672-4 . 26907711 10.1007/s00261-016-0672-4

[21] Prassopoulos P, Daskalogiannaki M, Raissaki M, Hatjidakis A, Gourtsoyiannis N. Determination of normal splenic volume on computed tomography in relation to age, gender and body habitus. Eur Radiol. 1997;7(2):246–8. Epub 1997/01/01. 10.1007/s003300050145 . 9038125 10.1007/s003300050145

[22] Angulo P, Hui JM, Marchesini G, Bugianesi E, George J, Farrell GC, et al The NAFLD fibrosis score: a noninvasive system that identifies liver fibrosis in patients with NAFLD. Hepatology. 2007;45(4):846–54. Epub 2007/03/30. 10.1002/hep.21496 . 17393509 10.1002/hep.21496

[23] Treeprasertsuk S, Bjornsson E, Enders F, Suwanwalaikorn S, Lindor KD. NAFLD fibrosis score: a prognostic predictor for mortality and liver complications among NAFLD patients. World J Gastroenterol. 2013;19(8):1219–29. Epub 2013/03/14. 10.3748/wjg.v19.i8.1219 23482703 10.3748/wjg.v19.i8.1219 PMC3587478

[24] Sterling RK, Lissen E, Clumeck N, Sola R, Correa MC, Montaner J, et al Development of a simple noninvasive index to predict significant fibrosis in patients with HIV/HCV coinfection. Hepatology. 2006;43(6):1317–25. Epub 2006/05/27. 10.1002/hep.21178 . 16729309 10.1002/hep.21178

[25] Sanyal D, Mukherjee P, Raychaudhuri M, Ghosh S, Mukherjee S, Chowdhury S. Profile of liver enzymes in non-alcoholic fatty liver disease in patients with impaired glucose tolerance and newly detected untreated type 2 diabetes. Indian J Endocrinol Metab. 2015;19(5):597–601. Epub 2015/10/02. 10.4103/2230-8210.163172 26425466 10.4103/2230-8210.163172 PMC4566337

[26] Navaneethan U, Shen B. Hepatopancreatobiliary manifestations and complications associated with inflammatory bowel disease. Inflamm Bowel Dis. 2010;16(9):1598–619. Epub 2010/03/04. 10.1002/ibd.21219 . 20198712 10.1002/ibd.21219

[27] Margariti E, Deutsch M, Manolakopoulos S, Papatheodoridis GV. Non-alcoholic fatty liver disease may develop in individuals with normal body mass index. Ann Gastroenterol. 2012;25(1):45–51. Epub 2012/01/01. 24713801 PMC3959339

[28] Kumar R, Mohan S. Non-alcoholic Fatty Liver Disease in Lean Subjects: Characteristics and Implications. J Clin Transl Hepatol. 2017;5(3):216–23. Epub 2017/09/25. doi: 10.14218/JCTH.2016.00068 28936403 10.14218/JCTH.2016.00068 PMC5606968

[29] Moran C, Sheehan D, Shanahan F. The Changing Phenotype of Inflammatory Bowel Disease. Gastroenterol Res Pract. 2016;2016:1619053 Epub 2017/01/05. 10.1155/2016/1619053 28050166 10.1155/2016/1619053 PMC5168455

[30] Casanova MJ, Chaparro M, Molina B, Merino O, Batanero R, Duenas-Sadornil C, et al Prevalence of Malnutrition and Nutritional Characteristics of Patients With Inflammatory Bowel Disease. J Crohns Colitis. 2017;11(12):1430–9. Epub 2017/10/06. 10.1093/ecco-jcc/jjx102 . 28981652 10.1093/ecco-jcc/jjx102

[31] Sartini A, Gitto S, Bianchini M, Verga MC, Di Girolamo M, Bertani A, et al Non-alcoholic fatty liver disease phenotypes in patients with inflammatory bowel disease. Cell Death Dis. 2018;9(2):87 Epub 2018/01/26. 10.1038/s41419-017-0124-2 29367619 10.1038/s41419-017-0124-2 PMC5833704

[32] Bessissow T, Le NH, Rollet K, Afif W, Bitton A, Sebastiani G. Incidence and Predictors of Nonalcoholic Fatty Liver Disease by Serum Biomarkers in Patients with Inflammatory Bowel Disease. Inflamm Bowel Dis. 2016;22(8):1937–44. Epub 2016/07/06. 10.1097/MIB.0000000000000832 . 27379445 10.1097/MIB.0000000000000832

[33] Schoepfer AM, Vavricka S, Zahnd-Straumann N, Straumann A, Beglinger C. Monitoring inflammatory bowel disease activity: clinical activity is judged to be more relevant than endoscopic severity or biomarkers. J Crohns Colitis. 2012;6(4):412–8. Epub 2012/03/09. 10.1016/j.crohns.2011.09.008 . 22398068 10.1016/j.crohns.2011.09.008

[34] Anstee QM. Animal models in nonalcoholic steatohepatitis research: utility and clinical translation. Liver Int. 2011;31(4):440–2. Epub 2011/03/09. 10.1111/j.1478-3231.2011.02463.x . 21382155 10.1111/j.1478-3231.2011.02463.x

[35] Hebbard L, George J. Animal models of nonalcoholic fatty liver disease. Nat Rev Gastroenterol Hepatol. 2011;8(1):35–44. Epub 2010/12/02. 10.1038/nrgastro.2010.191 . 21119613 10.1038/nrgastro.2010.191

[36] Martinez LA, Larrieta E, Kershenobich D, Torre A. The Expression of PNPLA3 Polymorphism could be the Key for Severe Liver Disease in NAFLD in Hispanic Population. Ann Hepatol. 2017;16(6):909–15. Epub 2017/10/23. 10.5604/01.3001.0010.5282 . 29055919 10.5604/01.3001.0010.5282

[37] Hagstrom H, Nasr P, Ekstedt M, Hammar U, Stal P, Hultcrantz R, et al Risk for development of severe liver disease in lean patients with nonalcoholic fatty liver disease: A long-term follow-up study. Hepatol Commun. 2018;2(1):48–57. Epub 2018/02/07. 10.1002/hep4.1124 29404512 10.1002/hep4.1124 PMC5776871

[38] Hanachi M, Melchior JC, Crenn P. Hypertransaminasemia in severely malnourished adult anorexia nervosa patients: risk factors and evolution under enteral nutrition. Clin Nutr. 2013;32(3):391–5. Epub 2012/09/19. 10.1016/j.clnu.2012.08.020 . 22986227 10.1016/j.clnu.2012.08.020

[39] Rosen E, Sabel AL, Brinton JT, Catanach B, Gaudiani JL, Mehler PS. Liver dysfunction in patients with severe anorexia nervosa. Int J Eat Disord. 2016;49(2):151–8. Epub 2015/09/09. 10.1002/eat.22436 . 26346046 10.1002/eat.22436

[40] DeZwaan-McCabe D, Sheldon RD, Gorecki MC, Guo DF, Gansemer ER, Kaufman RJ, et al ER Stress Inhibits Liver Fatty Acid Oxidation while Unmitigated Stress Leads to Anorexia-Induced Lipolysis and Both Liver and Kidney Steatosis. Cell Rep. 2017;19(9):1794–806. Epub 2017/06/01. 10.1016/j.celrep.2017.05.020 28564599 10.1016/j.celrep.2017.05.020 PMC5520660

[41] Moller L, Stodkilde-Jorgensen H, Jensen FT, Jorgensen JO. Fasting in healthy subjects is associated with intrahepatic accumulation of lipids as assessed by 1H-magnetic resonance spectroscopy. Clin Sci (Lond). 2008;114(8):547–52. Epub 2007/11/10. 10.1042/CS20070217 . 17990983 10.1042/CS20070217

[42] Carr RM, Patel A, Bownik H, Oranu A, Kerner C, Praestgaard A, et al Intestinal Inflammation Does Not Predict Nonalcoholic Fatty Liver Disease Severity in Inflammatory Bowel Disease Patients. Dig Dis Sci. 2017;62(5):1354–61. Epub 2017/03/08. 10.1007/s10620-017-4495-0 . 28265826 10.1007/s10620-017-4495-0

[43] Principi M, Iannone A, Losurdo G, Mangia M, Shahini E, Albano F, et al Nonalcoholic Fatty Liver Disease in Inflammatory Bowel Disease: Prevalence and Risk Factors. Inflamm Bowel Dis. 2018;24(7):1589–96. Epub 2018/04/25. 10.1093/ibd/izy051 . 29688336 10.1093/ibd/izy051

[44] Glassner K, Malaty HM, Abraham BP. Epidemiology and Risk Factors of Nonalcoholic Fatty Liver Disease Among Patients with Inflammatory Bowel Disease. Inflamm Bowel Dis. 2017;23(6):998–1003. Epub 2017/05/17. 10.1097/MIB.0000000000001085 . 28511199 10.1097/MIB.0000000000001085

[45] Endo H, Niioka M, Kobayashi N, Tanaka M, Watanabe T. Butyrate-producing probiotics reduce nonalcoholic fatty liver disease progression in rats: new insight into the probiotics for the gut-liver axis. PLoS One. 2013;8(5):e63388 Epub 2013/05/23. 10.1371/journal.pone.0063388 23696823 10.1371/journal.pone.0063388 PMC3656030

[46] Cheng C, Tan J, Qian W, Zhang L, Hou X. Gut inflammation exacerbates hepatic injury in the high-fat diet induced NAFLD mouse: Attention to the gut-vascular barrier dysfunction. Life Sci. 2018;209:157–66. Epub 2018/08/11. 10.1016/j.lfs.2018.08.017 . 30096384 10.1016/j.lfs.2018.08.017

[47] Marra F, Svegliati-Baroni G. Lipotoxicity and the gut-liver axis in NASH pathogenesis. J Hepatol. 2018;68(2):280–95. Epub 2017/11/21. 10.1016/j.jhep.2017.11.014 . 29154964 10.1016/j.jhep.2017.11.014

[48] Miele L, Valenza V, La Torre G, Montalto M, Cammarota G, Ricci R, et al Increased intestinal permeability and tight junction alterations in nonalcoholic fatty liver disease. Hepatology. 2009;49(6):1877–87. Epub 2009/03/18. 10.1002/hep.22848 . 19291785 10.1002/hep.22848

[49] Siebler J, Galle PR, Weber MM. The gut-liver-axis: endotoxemia, inflammation, insulin resistance and NASH. J Hepatol. 2008;48(6):1032–4. Epub 2008/05/13. 10.1016/j.jhep.2008.03.007 . 18468548 10.1016/j.jhep.2008.03.007

[50] Hossain N, Afendy A, Stepanova M, Nader F, Srishord M, Rafiq N, et al Independent predictors of fibrosis in patients with nonalcoholic fatty liver disease. Clin Gastroenterol Hepatol. 2009;7(11):1224–9, 9.e1–2. Epub 2009/06/30. . 19559819 10.1016/j.cgh.2009.06.007

[51] Younossi ZM, Blissett D, Blissett R, Henry L, Stepanova M, Younossi Y, et al The economic and clinical burden of nonalcoholic fatty liver disease in the United States and Europe. Hepatology. 2016;64(5):1577–86. Epub 2016/10/22. 10.1002/hep.28785 . 27543837 10.1002/hep.28785

[52] European Association for the Study of the L, European Association for the Study of D, European Association for the Study of O. EASL-EASD-EASO Clinical Practice Guidelines for the management of non-alcoholic fatty liver disease. J Hepatol. 2016;64(6):1388–402. Epub 2016/04/12. 10.1016/j.jhep.2015.11.004 . 27062661 10.1016/j.jhep.2015.11.004

[53] Marin-Gabriel JC, Solis-Herruzo JA. Noninvasive assessment of liver fibrosis. Serum markers and transient elastography (FibroScan). Rev Esp Enferm Dig. 2009;101(11):787–99. Epub 2009/12/17. . 20001156 10.4321/s1130-01082009001100006

[54] McPherson S, Hardy T, Henderson E, Burt AD, Day CP, Anstee QM. Evidence of NAFLD progression from steatosis to fibrosing-steatohepatitis using paired biopsies: implications for prognosis and clinical management. J Hepatol. 2015;62(5):1148–55. Epub 2014/12/06. . 25477264 10.1016/j.jhep.2014.11.034

[55] Dela Cruz AC, Bugianesi E, George J, Day CP, Liaquat H, Charatcharoenwitthaya P, et al Characteristics and Long-Term Prognosis of Lean Patients With Nonalcoholic Fatty Liver Disease. Gastroenterology. 2014;146(5):S909–S.

[56] Yamamoto-Furusho JK, Sanchez-Osorio M, Uribe M. Prevalence and factors associated with the presence of abnormal function liver tests in patients with ulcerative colitis. Ann Hepatol. 2010;9(4):397–401. Epub 2010/11/09. . 21057158

[57] Lee SS, Park SH. Radiologic evaluation of nonalcoholic fatty liver disease. World J Gastroenterol. 2014;20(23):7392–402. Epub 2014/06/27. 10.3748/wjg.v20.i23.7392 24966609 10.3748/wjg.v20.i23.7392 PMC4064084

[58] Lee SS, Park SH, Kim HJ, Kim SY, Kim MY, Kim DY, et al Non-invasive assessment of hepatic steatosis: prospective comparison of the accuracy of imaging examinations. J Hepatol. 2010;52(4):579–85. Epub 2010/02/27. 10.1016/j.jhep.2010.01.008 . 20185194 10.1016/j.jhep.2010.01.008

[59] van Werven JR, Marsman HA, Nederveen AJ, Smits NJ, ten Kate FJ, van Gulik TM, et al Assessment of hepatic steatosis in patients undergoing liver resection: comparison of US, CT, T1-weighted dual-echo MR imaging, and point-resolved 1H MR spectroscopy. Radiology. 2010;256(1):159–68. Epub 2010/06/25. 10.1148/radiol.10091790 . 20574093 10.1148/radiol.10091790

[60] Shyangdan D, Clar C, Ghouri N, Henderson R, Gurung T, Preiss D, et al Insulin sensitisers in the treatment of non-alcoholic fatty liver disease: a systematic review. Health Technol Assess. 2011;15(38):1–110. Epub 2011/11/09. 10.3310/hta15380 22059955 10.3310/hta15380 PMC4781160

[61] Artz NS, Haufe WM, Hooker CA, Hamilton G, Wolfson T, Campos GM, et al Reproducibility of MR-based liver fat quantification across field strength: Same-day comparison between 1.5T and 3T in obese subjects. J Magn Reson Imaging. 2015;42(3):811–7. Epub 2015/01/27. 10.1002/jmri.24842 25620624 10.1002/jmri.24842 PMC4803480

[62] Kang GH, Cruite I, Shiehmorteza M, Wolfson T, Gamst AC, Hamilton G, et al Reproducibility of MRI-determined proton density fat fraction across two different MR scanner platforms. J Magn Reson Imaging. 2011;34(4):928–34. Epub 2011/07/20. 10.1002/jmri.22701 21769986 10.1002/jmri.22701 PMC4803481

[63] Neuschwander-Tetri BA, Caldwell SH. Nonalcoholic steatohepatitis: summary of an AASLD Single Topic Conference. Hepatology. 2003;37(5):1202–19. Epub 2003/04/30. 10.1053/jhep.2003.50193 . 12717402 10.1053/jhep.2003.50193

